# Development of a Cryopreservation Technique for Xenogeneic Kidney Grafts: Evaluation Using a Mouse Model

**DOI:** 10.3390/jcm11237237

**Published:** 2022-12-06

**Authors:** Tsuyoshi Takamura, Hiroshi Nagashima, Hitomi Matsunari, Shuichiro Yamanaka, Yatsumu Saito, Yoshitaka Kinoshita, Toshinari Fujimoto, Kei Matsumoto, Kazuaki Nakano, Hirotaka James Okano, Eiji Kobayashi, Takashi Yokoo

**Affiliations:** 1 Division of Nephrology and Hypertension, Department of Internal Medicine, The Jikei University School of Medicine, Tokyo 105-8461, Japan; 2 Meiji University International Institute for Bio-Resource Research, Kawasaki 214-8571, Japan; 3 PorMedTec Co., Ltd., Kawasaki 214-0034, Japan; 4 Department of Urology, Graduate School of Medicine, The University of Tokyo, Tokyo 113-8654, Japan; 5 Division of Regenerative Medicine, The Jikei University School of Medicine, Tokyo 105-8461, Japan; 6 Department of Kidney Regenerative Medicine, The Jikei University School of Medicine, Tokyo 105-8461, Japan

**Keywords:** kidney regeneration, cryopreservation, metanephros

## Abstract

To align the xeno-metanephros and renal progenitor cell timing for transplantation treatments, cryopreservation techniques and an efficient transportation of regenerated renal products such as xeno-metanephroi and renal progenitor cells should be established. Therefore, we propose a novel method of xenogeneic regenerative medicine for patients with chronic kidney disease by grafting porcine fetal kidneys injected with human renal progenitor cells. To develop a useful cryopreserve system of porcine fetal kidney and human renal progenitor cells, we examined the cryopreservation of a fetal kidney implanted with renal progenitor cells in a mouse model. First, we developed a new method for direct cell injection under the capsule of the metanephros using gelatin as a support for unzipped fetal kidneys. Then, we confirmed in vitro that the nephrons derived from the transplanted cells were regenerated even after cryopreservation before and after cell transplantation. Furthermore, the cryopreserved chimeric metanephroi grew, and regenerated nephrons were observed in NOD. We confirmed that even in cryopreserved chimeric metanephroi, transplanted cell-derived nephrons as well as fresh transplants grew.

## 1. Introduction

In recent years, the etiological analysis of chronic kidney disease (CKD) has progressed, but CKD still has high morbidity rates and is associated with a high risk of developing end-stage renal disease [1]. The current CKD treatment approaches suppress disease progression and prevent complications such as cardiovascular events, but no treatments can directly improve the renal function. Even if end-stage renal failure occurs, individuals can live with renal replacement therapy such as dialysis, but their quality of life is greatly restricted, and they incur huge medical expenses due to dialysis [2]. Kidney transplantation is the only way to treat renal failure, but few individuals benefit from the procedure due to a severe organ shortage [3]. Recent advances in porcine gene modification technology and immunosuppressive drugs have focused on xenogeneic kidney transplantation [4,5]. However, issues with xenogeneic cells in hosts under potent immunosuppressive drug administration remain unresolved, while comparisons with hemodialysis and quality of life improvements are unclear.

Instead of xenotransplantation, where organs from xenogeneic animals are directly transplanted, we developed chimeric kidneys as a novel treatment strategy. Specifically, human-derived renal progenitor cells were injected into a porcine metanephros to function as scaffolds, and new human-derived nephrons were generated in the body [6]. In this method, heterogeneous porcine metanephroi were used without vascular anastomosis in the recipient; the host blood vessels entered the porcine metanephroi that became chimeras with the human type, thereby weakening the rejection responses during developmental processes [7,8].

In our previous study, we increased the chimerism rate of host-type cells by measuring genetic alterations that facilitated the drug-induced elimination of nephron progenitor cells (NPCs) [9,10,11,12,13]. Furthermore, methods inducing NPCs from human induced pluripotent stem cells (iPS) have been established in recent years, and it is theoretically possible to regenerate kidneys with their own nephrons if NPCs are derived from iPS cells of patients with CKD [14,15,16].

Owing to these developments, heterogeneous renal regenerative medicine is being challenged. When providing human regenerated kidneys for clinical practice, patients must be in a transplantable condition, and xeno-metanephroi and renal progenitor cells derived from patient iPS cells must be supplied simultaneously. To align the xeno-metanephros and renal progenitor cell timing for transplantation treatments, cryopreservation techniques and an efficient transportation of regenerated renal products such as xeno-metanephroi and renal progenitor cells should be established.

In this study, we preclinically verified a cryopreservation system for porcine metanephroi and human renal progenitor cells. We investigated a cryopreservation method for metanephroi, renal progenitor cells, and a complex tissue implanted with renal progenitor cells into the metanephroi using a mouse model. We previously reported the cryopreservation of porcine metanephroi alone; thus, based on a cell transplantation method for the metanephros alone, we validated our cryopreservation protocol during the clinically assumed process from the outpatient stage to the infusion of renal progenitor cells into the metanephros [17]. We first developed techniques to compensate for metanephros fragility after freezing and thawing and extensively implanted exogenous cells under the renal capsule. We compared and verified the direct cell transplantation under the metanephric capsule using gelatin as a support (new method) by transplanting cells through the metanephric tissue under the capsule on the contralateral side (conventional method). We verified in vitro and in vivo that the nephrons derived from the transplanted cells regenerated even after cryopreservation before and after cell transplantation.

## 2. Materials and Methods

### 2.1. Animals

C57BL/6-Tg (CAG-EGFP) mice (green fluorescent protein [GFP] mice) were purchased from Sankyo Labo Service Japan. NOD/Shi-Scid IL-2RγKOJic (NOG) mice were purchased from CLEA Japan. Genetically modified B6;129-Gt(ROSA)26Sor[tm1(DTA)Mrc]/J (DTA), and B6;129-Six2[tm3(EGFP/cre/ERT2)Amc]/J (Six2-GCE) mice were purchased from The Jackson Laboratory. The Six2-GCE mice were crossed with the DTA mice to generate Six2-GCE+/DTA+ offspring. Mating was timed using 10–25-week-old mice. At 12:00 p.m. on the day the vaginal plug was detected, this was set as 0.5 days after mating.

The mice were housed in a temperature- and humidity-controlled environment with a 12 h light–dark cycle. The animals were provided with standard laboratory chow and water. The animal studies were approved by the Institutional Animal Care and Use Committee and the Recombinant Gene Research Safety Committee of the Jikei University School of Medicine (permit numbers: 2018-066, R1-1, and II-1-51). The studies conformed to the National Institutes of Health Guidelines for the Care and Use of Laboratory Animals. All efforts were made to minimize animal suffering.

### 2.2. Transplanted Cells

We used our previous method [18,19] that was based on a previous report [20]. Specifically, E14.5 GFP mice were euthanized by cervical dislocation, and their abdomens were opened to collect embryos. The mice were humanely euthanized by decapitation, and the metanephroi were isolated using micro-tweezers under a microscope and then stored on ice in Base Medium consisting of Modified Eagle Medium-α supplemented with 20% fetal bovine serum (SH30910, Hyclone, Logan, UT, USA) and 1% antibiotic–antimycotic (12561-056, Thermo Fisher Scientific, Waltham, MA, USA). After centrifuging at 700× *g* for 1 min to remove the supernatant, 1 mL of Accutase (AT104-500, Funakoshi, Tokyo, Japan) was added, and the suspension was incubated at 37 °C for 15 min, with manual pipetting every 5 min. The cells were then centrifuged at 300× *g* for 5 min, and the pellets were resuspended in 1 mL of phosphate-buffered saline (PBS) (045-29795, Wako, Osaka, Japan) and passed through a 40 μm cell strainer (352235, Corning, New York, NY, USA) after the manual pipetting. The filtrate was centrifuged at 300× *g* for 5 min. After completely removing the supernatant, the pellet was mixed gently by tapping the tube, and the suspension was incubated on ice until transplantation.

### 2.3. Cryopreservation

#### 2.3.1. Renal Progenitor Cell Cryopreservation

Transplanted cell cryopreservation was performed using the method by Nakagawa et al. [21]. In practice, the cell pellets separated for transplantation were suspended in 500 µL of STEM-CELLBANKER^®^ Dimethyl Sulfoxide (DMSO) Free GMP grade (CB063, Takara Bio, Tokyo, Japan) and added to the cryotubes. The tubes were placed in a BICELL^®^ container (BICELL, Funakoshi, Tokyo, Japan), stored overnight in a −80 °C freezer, and submerged in liquid nitrogen. The tubes were thawed by briefly placing the tube in a hot bath at 40 °C, adding 10 mL of Base Medium, pipetting, centrifuging at 300× *g* for 5 min, and removing the supernatant.

#### 2.3.2. Fetal Kidney Tissue Cryopreservation

Tissue freezing was performed by modifying previously reported methods [17,22]. The fetal metanephroi were cryopreserved using the vitrification method, with or without cell transplantation. First, the metanephroi were equilibrated in 7.5% ethylene glycol (EG) (055-00996, Wako, Osaka, Japan) and 7.5% DMSO (317275-100ML, Millipore, Burlington, MA, USA) in Base Medium on ice for 15 min and then soaked in 15% EG and 15% DMSO in Base Medium on ice for an additional 15 min. The metanephroi were then placed in a Cryotop (81111, Kitazato, Tokyo, Japan) and directly submerged in liquid nitrogen. When thawing, the cryotop was quickly transferred to Base Medium plus 1 M sucrose and immersed for 1 min at 42 °C, transferred to Base Medium plus 0.5 M sucrose and placed at room temperature for 3 min, and finally washed twice in Base Medium for 5 min each at room temperature. All cryopreserved metanephroi were thawed within 4 weeks of freezing and used in experiments.

### 2.4. Cell Injection

We used a new renal progenitor cell injection method in the fetal kidney capsule after freezing and thawing. The metanephroi were harvested from E14.5 Six2-GCE+/DTA+ mice, as we did from the GFP mice. Drops (10 µL) of 16.6% gelatin (G2500-100G, Sigma-Aldrich, Tokyo, Japan) in PBS at 40 °C were placed in a 10 cm dish (GD90-15, AS ONE, Osaka, Japan) at 40 °C. The metanephroi were encapsulated in the drops, and the gelatin allowed to solidify on ice. Subsequently, using an injection holder (HI-7, Narishige, Tokyo, Japan), transplanted cells prepared as mentioned above were introduced by passing through a glass needle (SK-IJ ID40, Sankyo Medic, Shizuoka, Japan; inner diameter = 40 µm). Under fluorescence stereomicroscopy (Leica M205FA), a glass needle was horizontally inserted into the capsular surface of the metanephros, and the cells were injected just below the capsular surface using a mouth pipette.

The injection method developed in this study was compared with a method we previously used. In the conventional method, the cells were injected in the same manner as in previous studies [11,12]. In brief, using fetal tissue as a base, a glass needle was inserted from the renal hilum through the renal parenchyma, and the cells were injected below the renal capsular surface with a mouth pipette. For semi-quantifying the injected cells, the same glass needle (inner diameter = 40 µm) was used, and the cells were transplanted by aspirating up to 1 mm from the tip of the glass needle using a mouth pipette. The cell-injected metanephroi were used for organ culture or sub-retroperitoneal transplantation into NOG mice.

### 2.5. Organ Culture

Organ cultures were performed as previously reported [18]. Cell-injected metanephroi were placed at the air–fluid interface of a polycarbonate filter (3401, Corning, New York, NY, USA; average pore size = 0.4 μm). The Base Medium supplemented with 2 ng/mL of 4OH-tamoxifen (H7904, Sigma-Aldrich, Tokyo, Japan) was changed every day, and the cells were harvested after culturing at 37 °C in 5% CO_2_ for 5 days.

### 2.6. Transplantation of Metanephros

Adult NOG mice were anesthetized with isoflurane (2817774, Pfizer, Tokyo, Japan) at an inhalation rate of 1–4 L/min, and laparotomy was performed through a midline abdominal incision. A pocket was created in the retroperitoneum of the descending aorta using micro-tweezers under a surgical microscope (Leica S9D). The metanephroi were transplanted into the pocket, and the operation was completed by closing the abdomen. Then, 200 mg/kg/day of tamoxifen, adjusted to 40 mg/mL in corn oil (032-17016, Wako, Osaka, Japan), was administered by gavage from the day before transplantation for 6 days. Two weeks after transplantation, the mice were humanely euthanized by cervical dislocation, and the transplanted metanephroi were harvested.

### 2.7. Whole-Mount Immunostaining

The organo-cultured metanephroi were fixed in 4% paraformaldehyde (163-20145, Wako, Osaka, Japan) at 4 °C for 30 min and washed three times in PBS. The samples were blocked in PBS plus 1% donkey serum (017-000-001, Jackson ImmunoResearch Laboratories, West Groove, PA, USA), 0.2% skim milk (190-12865, Wako, Osaka, Japan), and 0.3% Triton X (25987-85, Nacalai Tesque, Tokyo, Japan) at room temperature for 1 h. After blocking, the samples were incubated overnight at 4 °C with a primary antibody, washed three times in PBS, and incubated with Alexa Fluor 488 (A-21131, Thermo Fisher Scientific), Alexa Fluor 555 (A-31572, Thermo Fisher Scientific), and Alexa Fluor 650 (SA5-10029, Thermo Fisher Scientific) for 1 h at room temperature. Subsequently, sthe amples were washed three times in PBS and mounted in mounting medium (S36972, Thermo Fisher Scientific) plus 4′6-diamidino-2-phenylindole (DAPI) (P36931, Thermo Fisher Scientific). The samples were evaluated under a fluorescence microscope (LSM880 Carl Zeiss, Oberkochen, Germany).

### 2.8. Hematoxylin-and-Eosin Staining and Immunostaining of Frozen Sections

Metanephroi from NOG mice were fixed in 4% formaldehyde at 4 °C for 30 min and dehydrated overnight in PBS plus 20% sucrose. Then, the samples were embedded in OCT compound (4583, Sakura Finetechnical, Tokyo, Japan) and frozen, and 10 μm sections were prepared. Hematoxylin-and-eosin staining was performed according to the standard histological procedures. Before immunostaining, the samples were washed three times in PBS and incubated with citrate buffer at 120 °C for 10 min to activate the antigens. After washing three times in PBS, blocking was performed at room temperature for 1 h, similar to what we did for whole-mount staining. Subsequently, the samples were incubated overnight at 4 °C with a primary antibody, washed three times in PBS, and incubated with a secondary antibody at room temperature for 1 h. The samples were washed three times in PBS and mounted in mounting medium plus DAPI. All samples were evaluated under a fluorescence microscope (LSM880 Carl Zeiss).

### 2.9. Cap Mesenchyme Counting

Whole samples were imaged under a fluorescence microscope. A GFP- (ab13970, Abcam, Tokyo, Japan), Six2- (11562-1-AP, Proteintech, Tokyo, Japan) co-positive cell cluster attached around CK8- (TROMA-I-C, Developmental Studies Hybridoma Bank, Iowa City, IA, USA) positive cellswas defined as one transplanted cell-derived cap mesenchyme (CM), and the total number was counted. At least three samples were measured for each condition.

## 3. Results

### 3.1. Experiment 1: Comparing the Transplantation Efficiency Using Different Cell Transplantation Methods

**Figure 1:**
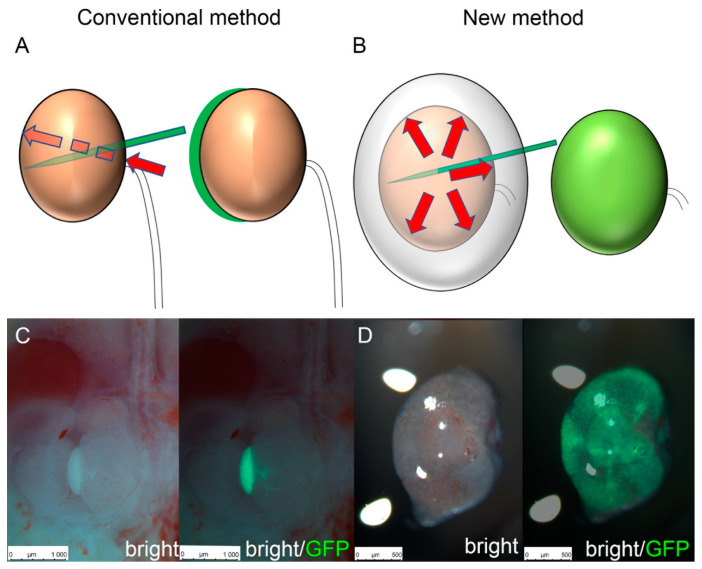
Schematic of cell transplantation methods. (**A**) Schematic of the conventional method. It is necessary to penetrate the renal parenchyma from the renal hilum. (**B**) Schematic of the new method. It can transplant cells into any renal capsule. (**C**) Representative fluorescence stereomicroscopy images of cell transplantation by the conventional method. Mouse Green fluorescent protein (GFP) renal progenitor cells were injected into an E14.5 Six2-GCE+/diphtheria toxin fragment A+ metanephros. (**D**) Representative fluorescence stereomicroscopy images of cells transplanted by the new method.

**Figure 2:**
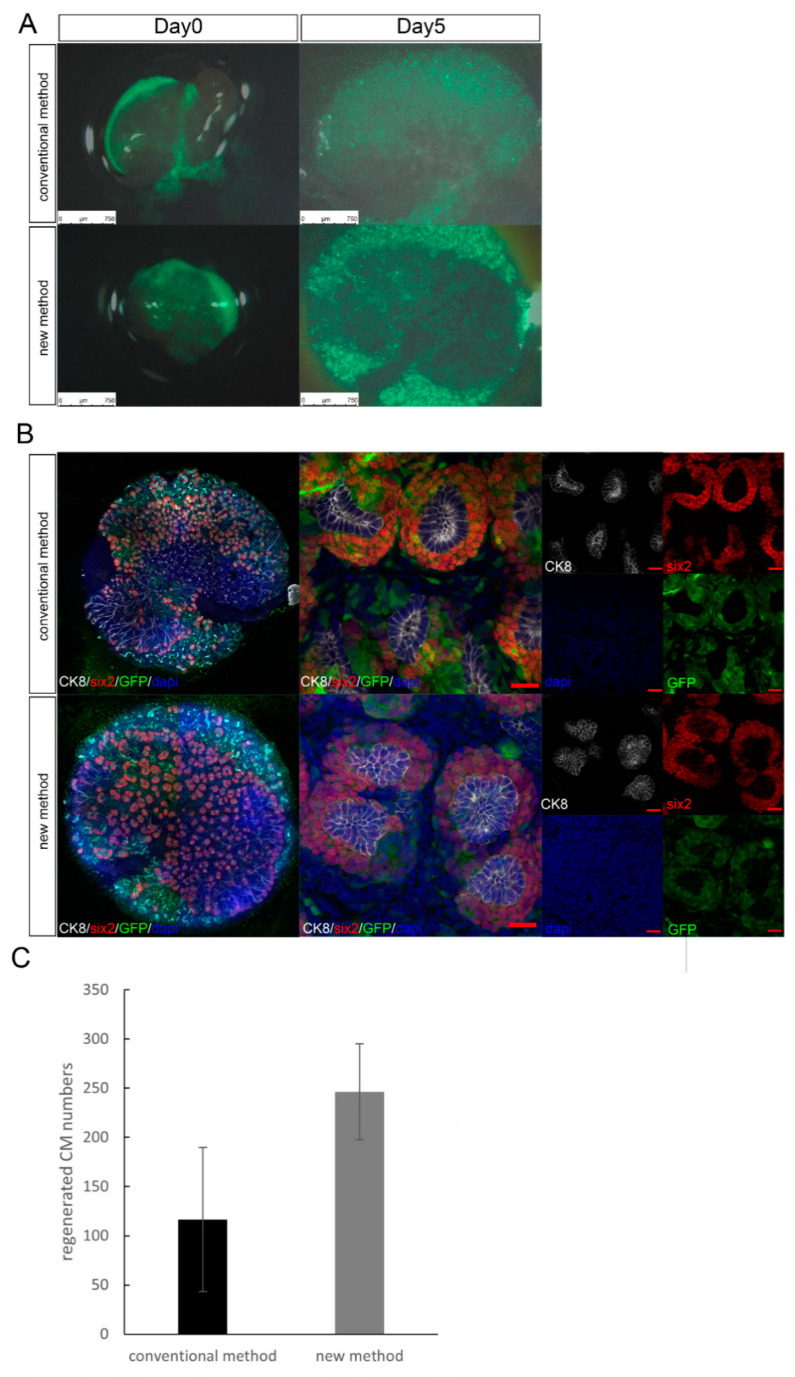
Differences in cell distribution for each cell transplantation method. (**A**) Organ culture of E14.5 Six2-GCE+/diphtheria toxin fragment A+ mouse metanephroi. Mouse green fluorescent protein (GFP) renal progenitor cells were injected into the metanephroi by each method and administrated 4OH-tamoxifen. (**B**) Immunostaining of the metanephroi in (**A**). In the new method, transplanted cell-derived cap mesenchymes (CMs) were distributed practically throughout the metanephros, including the peripheral and central parts of the renal pelvis. (scale bar, 20 µm). (**C**) Measurement of the transplanted cell-derived CMs. Error bars in bar plots represent standard errors of the mean. DAPI, 4′6-diamidino-2-phenylindole; CK8, cytokeratin 8.

Thus far, cell injection into heterologous metanephroi has been performed primarily in fresh or short-term storage samples. In this study, we first reduced needle penetration damage by fixing the fetal kidney tissue capsule and improving tissue fragility with gelatin (Figure 1). The following steps were performed to demonstrate injury reduction. We examined if nephrons were regenerated using the new cell transplantation method such that they could be transplanted into cryopreserved metanephroi alone. We performed maximal cell transplantation into Six2-GCE+/DTA+ mice metanephroi using the conventional and the new transplantation methods, followed by in vitro organ culture. In our transplantation method, unlike the conventional one, there was no need to penetrate the renal parenchyma from the renal hilum; thus, it was possible to transplant the cells into a wide area under any renal capsule (Video S1). In organ culture evaluation, the transplanted cell-derived CMs were distributed practically throughout the metanephroi, including the peripheral and central parts of the renal pelvis. These were previously difficult to transplant using conventional methods. In our method, the number of regenerated CM was higher than that obtained with the conventional method (Figure 2).

### 3.2. Experiment 2: Comparing the Transplantation Efficiency Using Different Cryopreserving Timings

**Figure 3:**
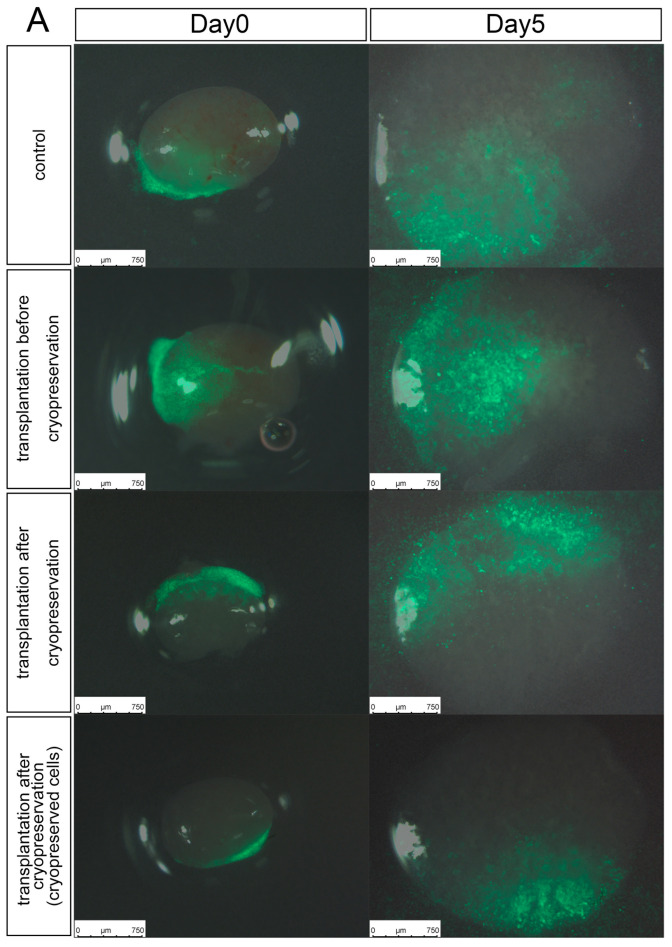

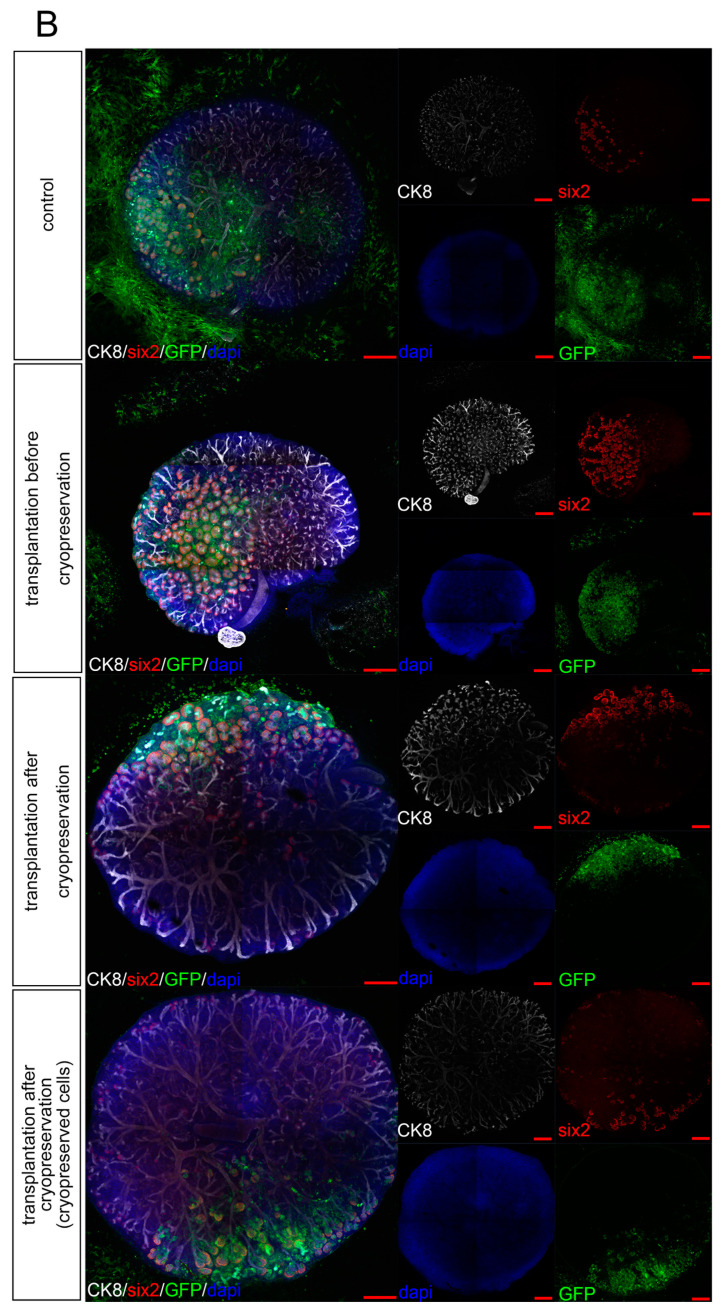

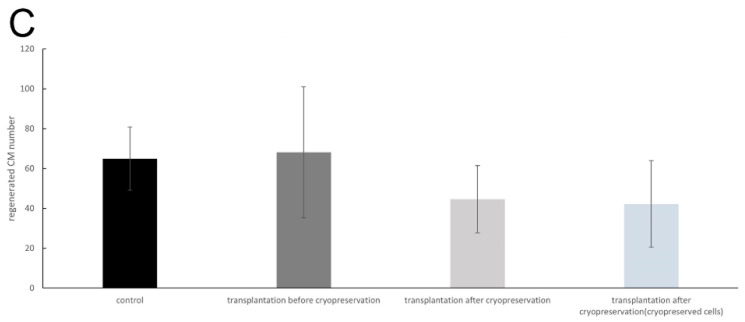
In vitro regeneration of nephrons at different cryopreservation timings. (**A**) Organ culture of E14.5 Six2-GCE+/diphtheria toxin fragment A+ mouse metanephroi. Mouse green fluorescent protein (GFP) renal progenitor cells were injected into the metanephroi before or after cryopreservation. Cryopreserved GFP renal progenitor cells were also used when the metanephroi were injected after cryopreservation. (**B**) Immunostaining of the metanephroi in (**A**). Regardless of the cryopreservation timing, the transplanted cells engrafted and differentiated at the ureteric tip to form the cap mesenchyme (CM) (scale bar, 400 µm). (**C**) Measurement of transplanted cell-derived CMs. Error bars in bar plots represent standard errors of the mean. DAPI, 4′6-diamidino-2-phenylindole; CK8, cytokeratin 8.

We verified if cell transplantation and nephron regeneration were possible at arbitrary timings, even if the metanephroi were thawed. Using our transplantation method, six2-GCE +/DTA + mice metanephroi were transplanted, with a constant renal progenitor cell density. They were cryopreserved and thawed before and after transplantation. The number of CMs was evaluated after organ culture. Regardless of the cryopreservation timing, the transplanted cells engrafted and differentiated at the ureteric tip to form the CMs. We confirmed that the transplanted cells regenerated into nephrons even after cryopreservation using the new method (Figure 3). In addition, based on the CM numbers obtained with the transplanted cells, cell transplantation before cryopreservation had practically the same engraftment efficiency as the control albeit with greater error. Vitrification-frozen metanephric tissue received exogenous NPCs and served as a scaffold for new nephrons. In addition, it was possible to generate neo-nephrons by simultaneously vitrifying and thawing metanephroi and renal progenitor cells.

### 3.3. Experiment 3: In Vivo Function of Cryopreserved Cell-Implanted Metanephroi

**Figure 4:**
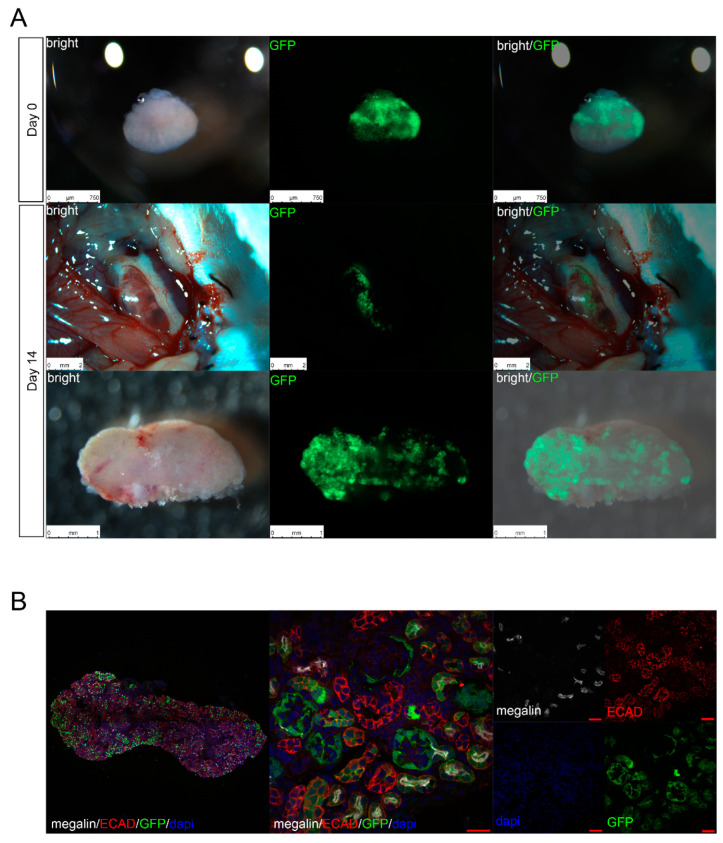
In Vivo regeneration of renal tissue using cryopreserved metanephroi. (**A**) Fluorescence stereomicroscopy images of cryopreserved E14.5 Six2-GCE+/diphtheria toxin fragment A+ mouse metanephroi injected with mouse green fluorescent protein (GFP) renal progenitor cells. The metanephroi were transplanted into NOD/Shi-Scid IL-2RγKOJic mice and were harvested two weeks after transplantation. (**B**) Immunostaining of frozen sections created from the collected specimens revealed GFP-positive neo-glomeruli which originated from transplanted mouse GFP-renal progenitor cells. Megalin-positive neo-proximal tubules and E-cadherin (ECAD)-positive neo-distal tubules can also be seen (scale bar, 20 µm). DAPI, 4′6-diamidino-2-phenylindole.

In previous experiments, we confirmed in vitro that metanephroi and transplanted cells regenerated nephrons from transplanted cells even after cryopreservation. To confirm tissue growth in vivo, cryopreserved metanephroi transplanted with renal progenitor cells were transplanted into the retroperitoneum of NOG mice. GFP+/megalin+ (610181, BD Biosciences, Franklin Lakes, NJ, USA) and GFP+/E-cadherin+ (SC-16478, Santa Cruz Biotechnology, Santa Cruz, CA, USA) cells were sporadically identified in metanephroi from NOG mice, and although NOG mice-derived cells remained, the transplanted cells differentiated into glomeruli and renal tubules (Figure 4). We found that 38 out of 64 glomeruli were chimerized, and the glomeruli generated from the transplanted cells contained NOG mice blood vessels and were integrated in the NOG mice’s circulatory system.

## 4. Discussion

To accomplish a xenogeneic renal regeneration strategy, we established an on-demand supply system for porcine metanephroi as scaffolds and renal progenitor cells that differentiated into a human kidney on the scaffold according to patient requirements. In this preclinical study, we used rodent metanephroi to extensively transplant renal progenitor cells into the metanephroi and examine if the metanephroi, which provided a developmental environment for the exogenous renal progenitor cells, could regenerate nephrons even under freeze–thaw stress.

We previously generated regenerative renal buds by injecting exogenous renal progenitor cells into metanephroi in rodent models [11,12,13]. Nephrogenesis occurs after NPCs are transplanted into the nephrogenic zone under the metanephric capsule, colonize the ureteric bud tip of the host, and reconstitute the CM. This reconstitution, which is termed progenitor cell replacement, requires a physical contact between the NPCs and the ureteric sprout tip that has been cleared of host cells. In other words, the host NPCs must be removed, and exogenous NPCs must be transplanted simultaneously over a wide area to facilitate their contact with vacant CM tips. However, in our conventional transplantation method, the insertional direction of the glass tube into the metanephroi was from the renal hilum, and the needle tip penetrated the renal parenchyma and passed under the renal capsule, from which the cells were transplanted into the nephrogenic zone. In this case, it was difficult to transplant the cells near the renal hilum because the direction and insertion angle of the needle were limited (Figure 1A,B). Furthermore, since the metanephroi moved during cell transplantation, we had to cut off the fetal dorsal region and transplant the cells while keeping the metanephroi attached to the fetus. Therefore, cell transplantation into fetal metanephroi was not possible [18]. In our new method, the metanephroi were fixed by solidified gelatin to restrict their movement, making cell transplantation feasible. Thus, the NPC-transplanted area and the contact area with vacant CMs increased. Therefore, we increased the CM number compared to that obtained with the conventional method (Figure 2).

Next, we developed frozen stocks to increase the regenerative renal bud availability for transplantation. Metanephroi as scaffolds are sufficiently small compared to adult kidneys and can be cryopreserved by vitrification [17]. However, the cryogen permeability and freeze–thaw rates are different between general organ tissues and single cells [23,24], and the effects of freezing regenerative renal buds containing both cells and metanephroi tissue are unknown. We showed that progenitor cell replacement occurred, and the CM was reconstituted even when regenerated kidney buds were frozen and thawed using the vitrification method. Unlike adult organs, the metanephroi are small and permeable to vitrification cryosolutions; therefore, there is little difference in cryosolution permeation between tissues and filled cells, thereby enabling the simultaneous vitrification and thawing of cells/tissues with different properties.

In addition, a successful post-transplantation of progenitor cells was observed even when unfrozen and slowly frozen NPCs were transplanted into frozen metanephroi (Figure 4). Although not a significant difference, a more extensive CM progenitor cell replacement was observed in cells transplanted before freezing than in cells transplanted after freezing. One reason for this difference was that, after freezing, the renal capsule became slightly fragile due to the exposure of the metanephroi to a high-osmotic-pressure cryosolution and liquid nitrogen. Hence, when injected under the capsule, cells might have been expelled from the outside. However, vitrification freezing of metanephroi alone has been performed thus far, and even if the renal capsule becomes slightly fragile, cell colonization can be improved by introducing a more elaborate transplantation method using a mechanical arm or an injector [25], instead of a manual operation.

When the vitrified regenerative renal buds were transplanted into the retroperitoneum of adult NOG mice using our new method, the recipient engraftment was similar to that reported in previous studies. Thus, the regenerative renal buds survived after freezing and thawing, and the transplanted progenitor cells differentiated into nephrons.

Our study has some limitations: the study period was short, and effects after long-term cryopreservation were not observed. In addition, verification in large animals such as pigs is required for clinical use, but currently, porcine models with a renal progenitor cell replacement system are still under development; therefore, freezing and thawing in porcine metanephroi cannot be verified yet. However, porcine metanephroi are similar to rodent metanephroi in size and can be vitrified and frozen; therefore, cryopreservation is feasible [26].

Extensive NPC transplantation may increase exogenous progenitor cells in CMs and increase the number of regenerative nephrons. In addition, we clarified that regenerative renal buds containing both NPCs and metanephroi generated chimeric nephrons even after vitrification. Furthermore, since fetal organs are small, frozen liquid permeability is high, and the effects of cooling and heating on the whole organ are unlikely to differ. The fact that the regenerative renal buds were cryopreserved is a distinct advantage of our organ regeneration technique using fetal organs and shows the possibility of using regenerative renal buds for heterogeneous regenerative medicine. In future studies, we will investigate large animals such as pigs that are similar to humans physiologically. We aim to stably supply human nephrons as replacement organs for renal failure by generating nephrons in heterologous metanephroi and storing them frozen.

## 5. Conclusions

It is possible to transplant a wide range of cells into mouse metanephroi. Freezing and thawing were possible before and after progenitor cell replacement. We showed that regenerative renal bud frozen stocks could reduce time and distance constraints of transplantation organs in the future. Our research not only provides useful information for the regeneration and clinical application of humanized kidneys but also can be used as a transplantation and preservation method for different microstructures.

## Data Availability

All relevant data supporting the findings of this study are either included within the article or are available upon request from the corresponding author.

## Supplementary Materials

The following supporting information can be downloaded at: https://www.mdpi.com/article/10.3390/jcm11237237/s1, Video S1: The new transplantation method.
